# Society Meetings

**Published:** 1905-06

**Authors:** 


### SOCIETY MEETINGS.

The National Association of United States Pension Examining Surgeons will hold its fourth annual meeting at Chicago, Friday and Saturday, July 7 and 8, 1905, under the following administration: president, Thomas D. Davis, Pittsburg; treasurer, Chas. H. Glidden, Little Falls, N. Y.; secretary, Wheelock Rider, Rochester.

---

The Buffalo Academy of Medicine held meetings during the month of May, 1905, as follows:

*Section on Surgery.*—Tuesday evening, May 2. Program: (a) Massage of the heart in cases of impending death, William C. Phelps; (b) Manifestations of lithemia in the spine and lower extremities, simulating orthopedic conditions, Prescott Le Breton. Election of officers for section.

*Section on Medicine.*—Tuesday evening, May 9, Program: (a) The nurse or the doctor, Albert T. Lytle; (b) Treatment of disease by means other than drugs, A. W. Bayliss. Election of officers for section.

*Section on Pathology.*—Tuesday evening, May 16. Program: Some experiments on rabbits in connection with the correction of spinal curvature, Prescott Le Breton. Election of officers for section.

*Section on Obstetrics.*—Tuesday evening, May 23. Program: Alcohol in puerperal sepsis, H. P. Jack, Canisteo. Election of officers for section.

